# Effects of Lewis Basicity and Acidity on σ-Hole Interactions in Carbon-Bearing Complexes: A Comparative Ab Initio Study

**DOI:** 10.3390/ijms232113023

**Published:** 2022-10-27

**Authors:** Mahmoud A. A. Ibrahim, Mohammed N. I. Shehata, Al-shimaa S. M. Rady, Hassan A. A. Abuelliel, Heba S. M. Abd Elhafez, Ahmed M. Shawky, Hesham Farouk Oraby, Tamer H. A. Hasanin, Mahmoud E. S. Soliman, Nayra A. M. Moussa

**Affiliations:** 1 Computational Chemistry Laboratory, Chemistry Department, Faculty of Science, Minia University, Minia 61519, Egypt; 2 School of Health Sciences, University of Kwa-Zulu-Natal, Westville, Durban 4000, South Africa; 3 Science and Technology Unit (STU), Umm Al-Qura University, Makkah 21955, Saudi Arabia; 4 Deanship of Scientific Research, Umm Al-Qura University, Makkah 21955, Saudi Arabia; 5 Department of Chemistry, College of Science, Jouf University, Sakaka P.O. Box 2014, Saudi Arabia; 6 Molecular Bio-Computation and Drug Design Research Laboratory, School of Health Sciences, University of Kwa-Zulu-Natal, Westville, Durban 4000, South Africa

**Keywords:** σ-hole interactions, tetrel bonding interactions, Lewis basicity, Lewis acidity, ab initio calculations

## Abstract

The effects of Lewis basicity and acidity on σ-hole interactions were investigated using two sets of carbon-containing complexes. In Set I, the effect of Lewis basicity was studied by substituting the X_3_/X atom(s) of the NC-C_6_H_2_-X_3_ and NCX Lewis bases (LB) with F, Cl, Br, or I. In Set II, the W-C-F_3_ and F-C-X_3_ (where X and W = F, Cl, Br, and I) molecules were utilized as Lewis acid (LA) centers. Concerning the Lewis basicity effect, higher negative interaction energies (*E*_int_) were observed for the F-C-F_3_∙∙∙NC-C_6_H_2_-X_3_ complexes compared with the F-C-F_3_∙∙∙NCX analogs. Moreover, significant *E*_int_ was recorded for Set I complexes, along with decreasing the electron-withdrawing power of the X_3_/X atom(s). Among Set I complexes, the highest negative *E*_int_ was ascribed to the F-C-F_3_∙∙∙NC-C_6_H_2_-I_3_ complex with a value of −1.23 kcal/mol. For Set II complexes, *E*_int_ values of F-C-X_3_ bearing complexes were noted within the −1.05 to −2.08 kcal/mol scope, while they ranged from −0.82 to −1.20 kcal/mol for the W-C-F_3_ analogs. However, *V*_s,max_ quantities exhibited higher values in the case of W-C-F_3_ molecules compared with F-C-X_3_; preferable negative *E*_int_ were ascribed to the F-C-X_3_ bearing complexes. These findings were delineated as a consequence of the promoted contributions of the X_3_ substituents. Dispersion forces (*E*_disp_) were identified as the dominant forces for these interactions. The obtained results provide a foundation for fields such as crystal engineering and supramolecular chemistry studies that focus on understanding the characteristics of carbon-bearing complexes.

## 1. Introduction

Noncovalent interactions are evoking resurgent interest owing to their ubiquitous contributions to several fields, including crystal materials [1,2], molecular recognition [3,4], chemical reactions [5,6], adsorption [7], and biological processes [8]. Accordingly, further understanding of the origin and nature of noncovalent interactions along with their impacts on controlling molecular systems is one of the metrics of progress in modern chemistry. σ-Hole interaction is a crucial point of concern in the scope of noncovalent interactions due to its significant roles in ligand-acceptor interactions [9,10], self-assembly [11], and anion recognition [12].

The concept of σ-hole [13,14,15], as proposed by Politzer et al., was initially introduced as an insight into the halogen bonding phenomenon [16]. Afterward, it was extended to a remarkable family of noncovalent interactions in which the elements of group IV–VII tend to interact with a nucleophile (i.e., π-system [17,18], anion [19,20], or radical [21]). According to the literature, it was reported that the resulting attractive forces mainly depend on a local electron depletion region around the tetrel [22,23,24], pnicogen [25,26,27], chalcogen [28,29,30], and halogen [31,32,33,34] atoms. This region is usually directed along the extension of the σ-bond, and is hence labeled as a σ-hole. From this perspective, the σ-hole magnitude was principally associated with the electronegativity of the σ-hole donors and the covalently bonded atoms [35,36]. σ-hole interactions can also be strengthened by increasing the Lewis basicity of the nucleophile [37]. 

Among such σ-hole-based interactions, tetrel bonding interactions have gathered immense attention from experimental [10,38] and theoretical [23,39,40] viewpoints. Tetrel bonding plays significant roles in catalysis [41,42], supramolecular chemistry [43,44], and biological processes [6]. Preliminary studies uncovered the inability of the tetrel-bearing molecules to interact with Lewis bases (LB), NH_3_ as an example, and form tetrel bonds [45,46]. Remarkably, the electrostatic potentiality of tetrel-bearing systems to participate in tetrel bonding interactions was investigated with the help of point-of-charge (PoC) [47], and these observations were then confirmed using real Lewis bases [48,49]. An up-to-date work addressed the occurrence of σ-hole interactions within tetrel-bearing molecule∙∙∙Lewis acid (LA) dimers [50]. In addition, Lewis base∙∙∙tetrel-bearing molecule∙∙∙Lewis base trimers were precisely characterized [51]. Moreover, the favorable ability of tetrel-bearing molecules to engage in like∙∙∙like and unlike interactions with other neutral [22,52] and anion [53] candidates was revealed. Further, the effect of tetrel atomic size and its substituents on the tetrel bonding interactions within W-T-X_3_∙∙∙LB complexes (where T and X/W were tetrels and halogens, respectively) were studied [47,50]. It was reported that the interaction energy became more favorable with increasing: (i) the tetrel atomic size (i.e., C < Si < Ge < Sn), (ii) electronegativity of W atom, and (iii) the atomic size of the X_3_ halogens. More recently, the impact of external electric field (EEF) on these interactions was precisely assessed [54]. The negatively-directed EEF was found to decrease the interaction energies unfavorably. In contrast, the positively-directed EEF showed preferential enhancement of these energies.

**Figure 1:**
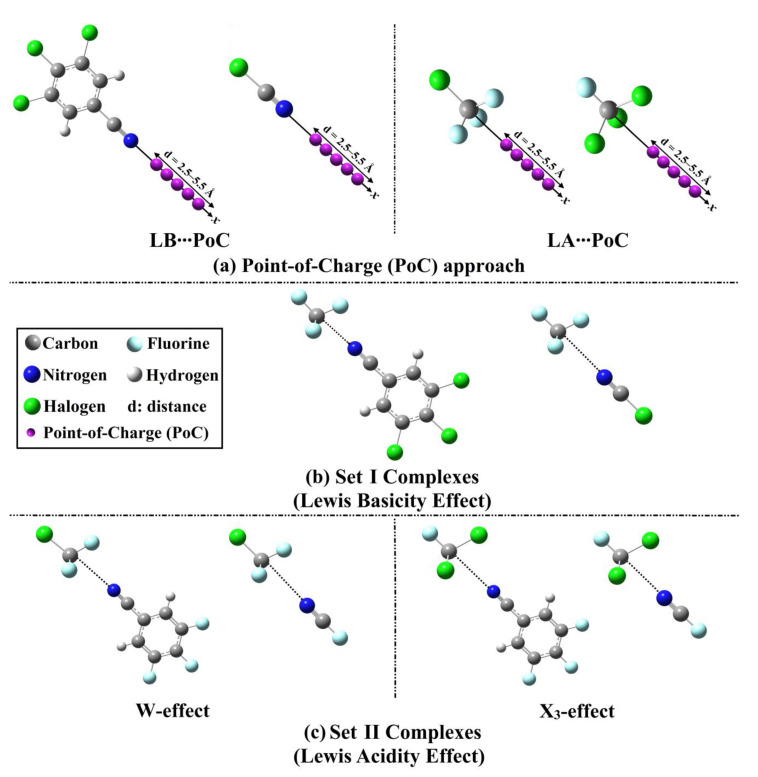
Illustrative representation for (**a**) the PoC calculations for Lewis base (LB)∙∙∙ and Lewis acid (LA)∙∙∙PoC systems, (**b**) Set I Complexes (F-C-F_3_∙∙∙NC-C_6_H_2_-X_3_/∙∙∙NCX), and (**c**) Set II Complexes (W-C-F_3_∙∙∙ and F-C-X_3_∙∙∙NC-C_6_H_2_-F_3_/NCF).

Indeed, tetrel bonding interactions are still an area of active research. In view of this, the presented study was designed to develop a comprehensive understanding of the effects of Lewis basicity and acidity on the interactions in carbon-bearing complexes (Figure 1). To pursue the aim of the current study, two sets of carbon-bearing complexes were investigated. In Set I complexes, the effect of Lewis basicity was thoroughly studied in F-C-F_3_∙∙∙NC-C_6_H_2_-X_3_/NCX (where X = F, Cl, Br, and I). In Set II complexes, the W-C-F_3_ and F-C-X_3_ (where X and W = F, Cl, Br, and I) molecules were utilized as Lewis acid (LA) centres to interact with NC-C_6_H_2_-F_3_/NCF as Lewis bases (LB). Geometrical optimization, electrostatic potential (EP) analyses, and point-of-charge (PoC) calculations were carried out for NC-C_6_H_2_-X_3_, NCX, W-C-F_3_, and F-C-X_3_ molecules. Interaction energy, quantum theory of atoms in molecules (QTAIM), and noncovalent interaction (NCI) index calculations were performed to uncover the strength and nature of the considered interactions. Symmetry-adapted perturbation theory (SAPT) analysis was also utilized to investigate the dominant forces within the studied interactions of the modeled complexes. The obtained findings would be informative for various ongoing works relevant to the scope of crystal engineering and materials science. 

## 2. Results and Discussion

### 2.1. Electrostatic Potential (EP) Analysis

**Figure 2:**
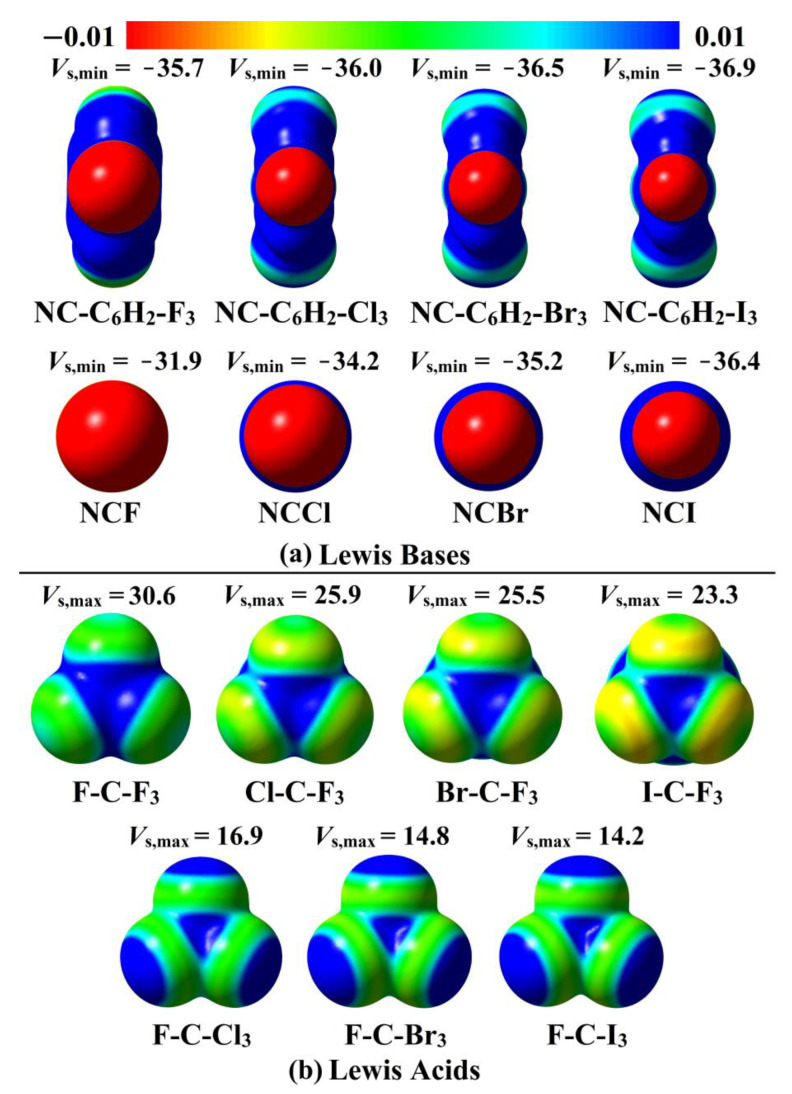
MEP maps of the utilized (**a**) NC-C_6_H_2_-X_3_ and NCX Lewis bases (LB) and (**b**) W-C-F_3_ and F-C-X_3_ Lewis acids (LA) molecules. The EP in these maps aligned within the −0.01 and +0.01 au range (red to blue colors). The *V*_s,min_/*V*_s,max_ values are in kcal/mol.

EP analysis was applied as an informative tool that gives qualitative and quantitative insights into the nucleophilic and electrophilic nature of the chemical systems [55,56]. Figure 2 involves molecular electrostatic potential (MEP) maps for all the investigated systems along with the values of the *V*_s,min_ (on NC-C_6_H_2_-X_3_ and NCX Lewis bases), and *V*_s,max_ (on W-C-F_3_ and F-C-X_3_ Lewis acids).

From MEP maps shown in Figure 2, observable red (i.e., negative EP) regions were denoted over the N atom surfaces of the inspected LBs, spotting the favorable potentiality of the explored molecules to interact with LAs attractively. In addition, the *V*_s,min_ values of the N atom (i.e., Lewis basicity) in the NC-C_6_H_2_-X_3_ and NCX molecules were found to decrease with decreasing the atomic size of the X_3_/X halogen(s) in the order of X = I > Br > Cl > F. For example, *V*_s,min_ exhibited values of −36.4, −35.2, −34.2, and −31.9 kcal/mol for the N atom of the NCI, NCBr, NCCl, and NCF molecules, respectively. It is also worth mentioning that the investigated NC-C_6_H_2_-X_3_ molecules demonstrated more negative *V*_s,min_ values than NCX analogs; for example, the *V*_s,min_ values were −36.9 and −36.4 kcal/mol for NC-C_6_H_2_-I_3_ and NCI, respectively. 

Passing to the inspected LA centres, notable positive EP regions (i.e., σ-hole) with different sizes were perceived (Figure 2). Evidently, σ-holes with more prominent sizes were observed within W-C-F_3_ molecules compared with the F-C-X_3_ counterparts, outlining the further ability of the former molecules to behave as carbon-bonding donors over the later ones. For instance, the *V*_s,max_ values of the I-C-F_3_ and F-C-I_3_ molecules were 23.3 and 14.2 kcal/mol, respectively. As evident, the σ-hole size increased with raising the electron-withdrawing power of the W/X_3_ halogen atom(s) that supported with appreciable *V*_s,max_ values in the case of fluorine-bearing molecules. Illustratively, *V*_s,max_ values were 23.3, 25.5, 25.9, and 30.6 kcal/mol for W-C-F_3_ molecules, as W = I, Br, Cl, and F, respectively.

### 2.2. Point-of-Charge (PoC) Calculations

**Figure 3:**
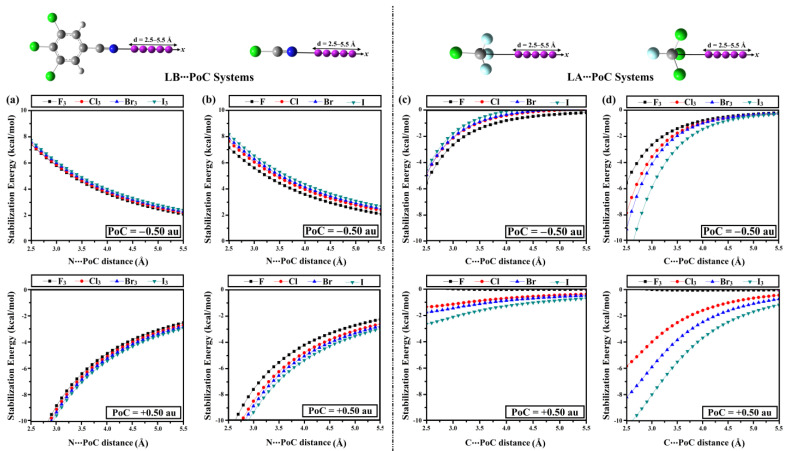
Stabilization/destabilization energy curve of the (**a**) NC-C_6_H_2_-X_3_∙∙∙, (**b**) NCX∙∙∙, (**c**) W-C-F_3_∙∙∙, and (**d**) F-C-X_3_∙∙∙PoC systems (where X and W = F, Cl, Br, and I) under the effect of PoC = ±0.50 au at N/C∙∙∙PoC distance ranging from 2.5 to 5.5 Å.

The PoC approach was recently reported as a trustworthy method to evaluate the σ- [17,57], π- [58,59], lp- [60], and R^•^ [61]-hole interactions from an electrostatic perspective. PoC calculations were conducted to inspect the distance impact on NC-C_6_H_2_-X_3_∙∙∙, NCX∙∙∙, W-C-F_3_∙∙∙, and F-C-X_3_∙∙∙PoC systems under the effect of PoC = ±0.50 au. Molecular energy curves were calculated for the investigated systems (Figure 3). Table 1 summarizes molecular destabilization and stabilization energies (*E*_destabilization_ and *E*_stabilization_) of the LB∙∙∙ and LA∙∙∙PoC systems under the effect of PoC = ±0.50 au at N/C∙∙∙PoC distance of 2.5 Å.

As shown in Figure 3, energetic destabilization and stabilization were observed to decrease along with increasing the Lewis base∙∙∙PoC intermolecular distance under the effect of negative and positive PoCs, respectively. Meanwhile, molecular stabilization energies were detected for all Lewis acid centres in the presence of negative and positive PoCs (Figure 3c,d). Generally, *E*_stabilization_ enlarged along with decreasing the Lewis acid∙∙∙PoC distance under the effect of negative and positive PoCs.

From Table 1, for all the NC-C_6_H_2_-X_3_∙∙∙ and NCX∙∙∙PoC systems, *E*_destabilization_ was found to increase as the *V*_s,min_ values increase under the effect of negative PoC. For instance, *E*_destabilization_ of NCF∙∙∙, NCCl∙∙∙, NCBr∙∙∙, and NCI∙∙∙^–^PoC systems were 7.13, 7.62, 7.85, and 8.15 kcal/mol along with *V*_s,min_ values of −31.9, −34.2, −35.2, and −36.4 kcal/mol for NCF∙∙∙, NCCl∙∙∙, NCBr∙∙∙, and NCI∙∙∙^–^PoC systems, respectively. Contrarily, under the effect of positive PoC, an inverse correlation was stated between the *E*_destabilization_ and the electron-withdrawing power of X_3_ and X halogen atom(s) of the NC-C_6_H_2_-X_3_ and NCX molecules, respectively. For instance, molecular stabilization energies (*E*_stabilization_) were −13.06, −13.39, −13.64, and −13.92 kcal/mol for NC-C_6_H_2_-F_3_∙∙∙, NC-C_6_H_2_-Cl_3_∙∙∙, NC-C_6_H_2_-Br_3_∙∙∙, and NC-C_6_H_2_-I_3_∙∙∙^+^PoC systems, respectively. Moreover, the NC-C_6_H_2_-X_3_ molecules were characterized by more favorable *E*_stabilization_ compared with the NCX ones that comply with *V*_s,min_ values. As an example, *E*_stabilization_ was −13.92 and −13.51 kcal/mol for NC-C_6_H_2_-I_3_∙∙∙ and NCI∙∙∙^+^PoC systems, accompanied by *V*_s,min_ values of −35.7 and −31.9 kcal/mol for NC-C_6_H_2_-F_3_ and NCF molecules, respectively. Turning to the W-C-F_3_∙∙∙ and F-C-X_3_∙∙∙PoC systems, the *E*_stabilization_ was observed to decrease in the order F-C-X_3_∙∙∙^–^PoC > F-C-X_3_∙∙∙^+^PoC > W-C-F_3_∙∙∙^–^PoC > W-C-F_3_∙∙∙^+^PoC. PoC findings highlighted the further ability of the F-C-X_3_ molecules to engage in tetrel bonding interactions compared with the W-C-F_3_ ones, which verified a reversed pattern with *V*_s,max_ values. 

### 2.3. Interaction Energy

**Figure 4:**
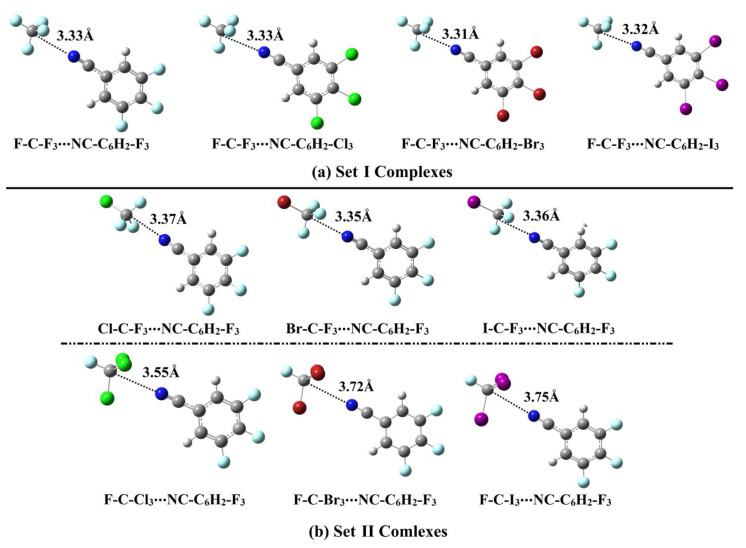
(**a**) Set I and (**b**) Set II complexes elucidating the Lewis basicity and acidity effects, respectively. The C∙∙∙N distances are evaluated in Å.

Set I and II complexes were utilized to study the effects of Lewis basicity and acidity on σ-hole interactions, respectively (see Figure 1). First, geometrical optimization for the investigated complexes was performed at MP2/aug-cc-pVTZ(PP) level of theory. The optimized structures of NC-C_6_H_2_-X_3_ containing complexes, along with their intermolecular distances, are displayed in Figure 4. As well, the NCX-containing complexes are given in Figure S1. Interaction energies (*E*_int_) were evaluated for Set I and II complexes at the same level of theory and are summerized in Table 2.

As shown in Table 2a, a progressive interaction energy pattern was noticed for the investigated Set I complexes along with increasing the atomic size of the X_3_/X halogen(s), as follows X = F < Cl < Br < I. This observation undoubtedly confirmed the contributions of the nucleophilic character of the Lewis bases (i.e., the effect of Lewis basicity) on σ-hole interactions within carbon-bearing complexes. For instance, the *E*_int_ were −1.16, −1.13, −1.13, and −1.05 kcal/mol for F-C-F_3_∙∙∙NCI, ∙∙∙NCBr, ∙∙∙NCCl, and ∙∙∙NCF complexes along with *V*_s,min_ values of −36.4, −35.2, −34.2, and −31.9 kcal/mol for the NCI, NCBr, NCCl, and NCF molecules, respectively.

Moreover, for Set I complexes, vast *E*_int_ were clearly seen in the case of F-C-F_3_∙∙∙NC-C_6_H_2_-X_3_ complexes, i.e., more than in their F-C-F_3_∙∙∙NCX counterparts, which is in line with *V*_s,min_ claims that showed higher negative EP regions for the former Lewis bases. For instance, *E*_int_ were −1.21 and −1.13 kcal/mol for the F-C-F_3_∙∙∙NC-C_6_H_2_-Br_3_ and ∙∙∙NCBr complexes alongside *V*_s,min_ values of −36.5 and −5.2 kcal/mol for NC-C_6_H_2_-Br_3_ and NCBr Lewis bases, respectively.

Interestingly, all Set II complexes showed negative *E*_int_ with different magnitudes, outlining the prominent impact of the Lewis acidity on the strength of carbon-bearing complexes. For Set II complexes, *E*_int_ of the W-C-F_3_∙∙∙Lewis base complexes were observed with lower negative values compared with the F-C-X_3_∙∙∙Lewis base analogs, in contrast with the results of the maximum positive EP regions (i.e., σ-hole). For example, from Table 2ii and Figure 2, *E*_int_ were −0.82 and −1.46 kcal/mol for the I-C-F_3_∙∙∙ and F-C-I_3_∙∙∙NCF complexes and accompanied by 23.3 and 14.2 kcal/mol *V*_s,max_ values for I-C-F_3_ and F-C-I_3_ molecules, respectively. This result was in agreement with the resurgent contributions of the X_3_ substituents (i.e., X_3_ = F_3_ < Cl_3_ < Br_3_ < I_3_) to the strength of the explored carbon-bearing complexes [50].

Comparatively, a linear correlation was found between *E*_int_ of W-C-F_3_ containing complexes and the corresponding σ-hole size, ensuring the attractive electrostatic interactions between the negative clouds of Lewis base and the positive ones of σ-hole [50]. For instance, *E*_int_ were –0.82, –0.91, –091, and –1.05 kcal/mol for I-C-F_3_∙∙∙, Br-C-F_3_∙∙∙, Cl-C-F_3_∙∙∙, and F-C-F_3_∙∙∙NCF complexes versus *V*_s,max_ values of 23.3, 25.5, 25.9, and 30.6 kcal/mol for I-C-F_3_, Br-C-F_3_, Cl-C-F_3_, and F-C-F_3_ molecules, respectively. Contrarily, the *E*_int_ of the F-C-X_3_∙∙∙Lewis base complexes was found to decrease with increasing the σ-hole size. For example, *E*_int_ of the F-C-X_3_∙∙∙NCF complexes were −1.05, −1.25, −1.25, and −1.46 kcal/mol versus *V*_s,max_ values of 30.6, 16.9, 14.8, and 14.2 kcal/mol, when X_3_ = F_3_, Cl_3_, Br_3_, and I_3_, respectively. Overall, the energetic features of Set II complexes were found to be consistent with PoC findings of W-C-F_3_/F-C-X_3_∙∙∙^–^PoC systems.

### 2.4. Quantum Theory of Atoms in Molecules (QTAIM) Analysis 

**Figure 5:**
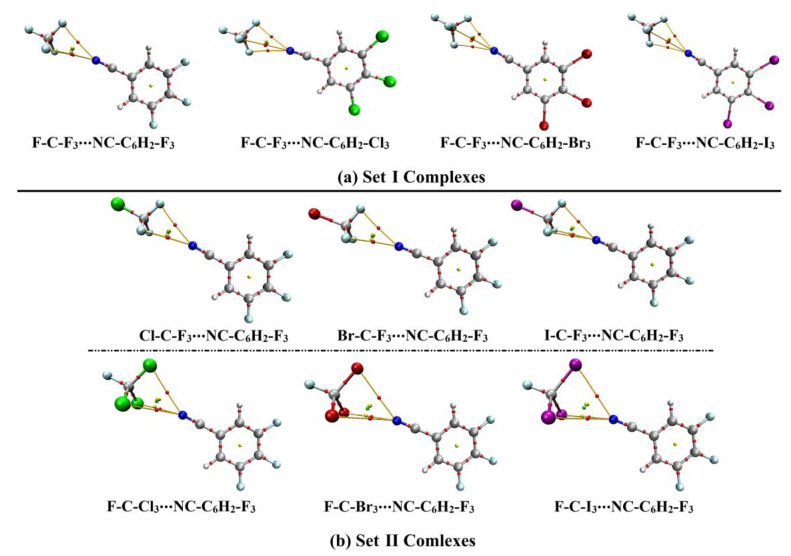
QTAIM diagrams of (**a**) Set I and (**b**) Set II complexes. Red dots point out the BCPs locations within the interacting species.

QTAIM analysis was performed to elucidate the origin of the intermolecular interaction [62]. QTAIM diagrams of the F-C-F_3_∙∙∙NC-C_6_H_2_-X_3_ and W-C-F_3_/F-C-X_3_∙∙∙NC-C_6_H_2_-F_3_ complexes are displayed in Figure 5. The corresponding diagrams for F-C-F_3_∙∙∙NCX and W-C-F_3_/F-C-X_3_∙∙∙NCF complexes are represented in Figure S3. The computed *ρ*_b_, ∇^2^*ρ*_b_, and H_b_ values are listed in Table 3.

From QTAIM diagrams demonstrated in Figure 5, three BPs and BCPs were denoted within the Set I and II complexes, highlighting the eminent contributions of the three coplanar halogens within the considered interactions that were in great agreement with previous studies [63]. The same observations were found for the F-C-F_3_∙∙∙NCX and W-C-F_3_/F-C-X_3_∙∙∙NCF complexes (Figure S3). As listed in Table 3, positive values of ∇^2^*ρ*_b_ and H_b_ accompanied by low values of *ρ*_b_ were obtained for all the studied complexes, asserting the closed-shell nature of the deemed interactions. For both the Set I and II complexes, the topological properties were remarked to be consistent with the interaction energy pattern.

Obviously, for Set I complexes, larger *ρ*_b_, ∇^2^*ρ*_b_, and H_b_ values were generally found for F-C-F_3_∙∙∙NC-C_6_H_2_-X_3_ complexes than their ∙∙∙NCX counterparts (Table 3). For example, ∇^2^*ρ*_b_ were 0.020980 and 0.020402 au for the F-C-F_3_∙∙∙NC-C_6_H_2_-Cl_3_ and ∙∙∙NCCl complexes, respectively. In general, *ρ*_b_, ∇^2^*ρ*_b_, and H_b_ values of the F-C-F_3_∙∙∙NC-C_6_H_2_-X_3_ and ∙∙∙NCX complexes were revealed to increase with decreasing the nucleophilicity of the interacted Lewis bases according to the following order X = I > Br > Cl > F. For instance, H_b_ of NCF, NCCl, NCBr, and NCI molecules were found with values of 0.001032, 0.001041, 0.001110, and 0.001111 au for F-C-F_3_∙∙∙NCF, ∙∙∙NCCl, ∙∙∙NCBr, and ∙∙∙NCI complexes, against *V*_s,min_ values of −31.9, −34.2, −35.2, and −36.4 kcal/mol, respectively.

Generally, for Set II complexes, the F-C-X_3_∙∙∙Lewis base complexes were characterized by higher *ρ*_b_, ∇^2^*ρ*_b_, and H_b_ values over the W-C-F_3_∙∙∙Lewis base counterparts, which coincided with the corresponding MP2 energetic features (Table 2). For example, H_b_ of I-C-F_3_∙∙∙ and F-C-I_3_∙∙∙NCF complexes were 0.000981 and 0.001017 au, along with *E*_int_ of −0.82 and −1.46 kcal/mol, respectively.

### 2.5. Noncovalent Interaction (NCI) Analysis

**Figure 6:**
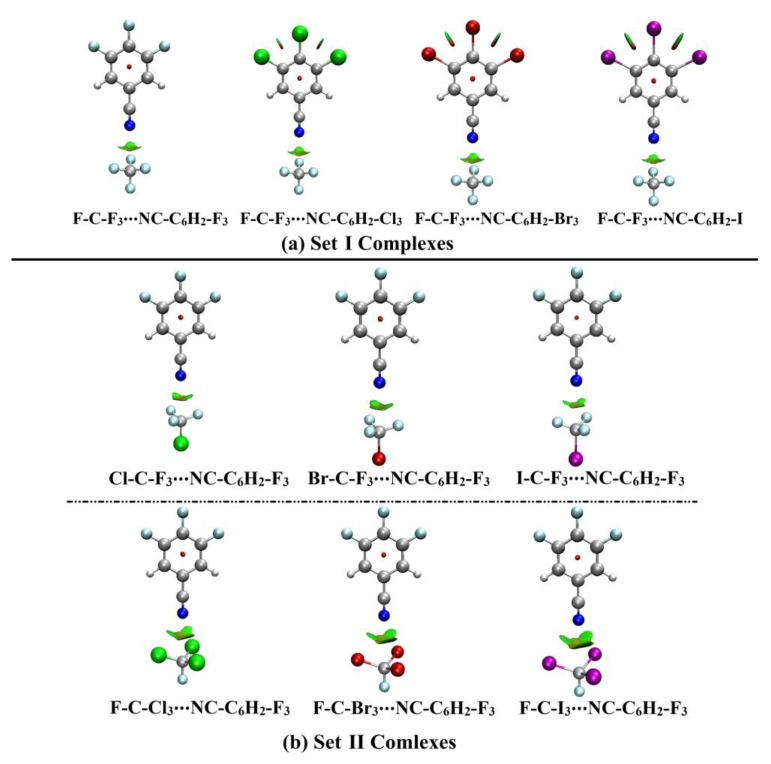
3D NCI plots of the (**a**) Set I and (**b**) Set II complexes. The color range extended based on the sign(λ_2_)*ρ* from −0.035 to 0.020 au (blue to red).

Following the announcement of Johnson et al. of the NCI index, the occurrence of inter- and intra-molecular interactions could be three-dimensionally recognized [64]. Subsequently, 2D and 3D NCI plots were generated with a 0.50 au reduced density gradient value. The color scale was in the range starting from blue (−0.035) to red (0.020) au. 3D NCI plots of F-C-F_3_∙∙∙NC-C_6_H_2_-X_3_ and W-C-F_3_/F-C-X_3_∙∙∙NC-C_6_H_2_-F_3_ complexes along with the F-C-F_3_∙∙∙NCX and W-C-F_3_/F-C-X_3_∙∙∙NCF analogs are illustrated in Figure 6 and Figure S4, respectively. Similarly, for the same pattern of complexes, 2D NCI-RDG plots were generated and are depicted in Figures S5 and S6.

As shown in Figure 6, the green surfaces between the interacting species outlined the weak attractive interactions within the Set I and II complexes. Further, the strength enhancement of the studied complexes was obviously noted by increasing the size of the obtained green regions (Figure 6 and Figure S4). Notably, the prominent role of the coplanar halogens was observed, ensuring the QTAIM findings. As can be seen from Figures S5 and S6, all the spikes were found with negative values of sign(λ_2_)*ρ*, affirming the occurrence of weak attractive interactions between the two interacting species.

### 2.6. Symmetry-Adapted Perturbation Theory (SAPT) Calculations 

**Figure 7:**
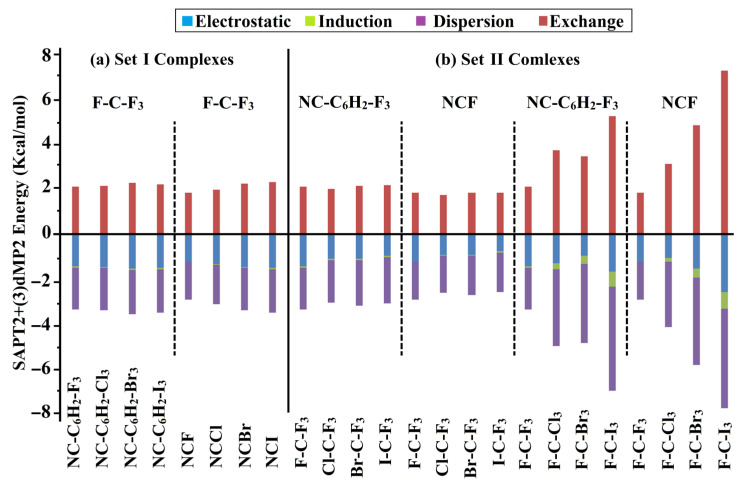
Bar chart illustrating the attractive forces against the repulsive ones of total SAPT2+(3)dMP2 energy for (a) Set I and (b) II complexes.

SAPT analysis was previously applied for its efficiency in analyzing the physical energetic components of noncovalent interactions [65]. Figure 7 represents the attractive forces versus the repulsive ones that were obtained from Total SAPT2+(3)dMP2 energies, revealing the most dominant energetic aspect within the interactions of Set I and II complexes. Table S1 lists the corresponding energetic values of such attractive and repulsive forces. 

As can be seen from Figure 7, negative energetic values were denoted for the electrostatic (*E*_elst_), induction (*E*_ind_), and dispersion energy (*E*_disp_) forces, outlining their contributions in stabilizing all the Set I and II complexes. Apparently, the *E*_disp_ was the most dominant force within such attractive forces. On the other hand, unfavorable contributions for the exchange energy (*E*_exch_) with positive values were also admitted. Illustratively, *E*_elst_, *E*_ind_, *E*_disp,_ and *E*_exch_ values were −2.66, −0.78, −5.24, and 7.53 kcal/mol for F-C-I_3_∙∙∙NC-C_6_H_2_-F_3_ complex (Table S1).

With respect to Set I complexes, the total attractive forces also affirmed the favorability of F-C-F_3_∙∙∙NC-C_6_H_2_-X_3_ complexes, with highly energetic features, over the F-C-F_3_∙∙∙NCX analogs. For Set II complexes, significant total attractive forces in the case of the F-C-X_3_ bearing complexes compared to W-C-F_3_ bearing complexes, which might be interpreted as a result of the resurgent contributions of X_3_ substituents. Generally, the total attractive forces (i.e., *E*_elst_, *E*_ind_, and *E*_disp_) were found to be consistent with the energetic features with some exceptions. Such exceptions might be interpreted by considering the *E*_exch_ contributions. Illustratively, the *E*_elst_/*E*_ind_/*E*_disp_/*E*_exch_ values of the F-C-F_3_∙∙∙NCF and F-C-Cl_3_∙∙∙NCF complexes were −1.26/−0.03/−1.73/1.91 and −1.09/−0.20/−3.00/3.32 kcal/mol, respectively.

## 3. Methods and Materials

In the current study, Set I and II complexes were used to investigating the effects of Lewis basicity and acidity on σ-hole interactions, respectively (see Figure 1). In that spirit, the F-C-F_3_∙∙∙NC-C_6_H_2_-X_3_/NCX and W-C-F_3_/F-C-X_3_∙∙∙NC-C_6_H_2_-F_3_/NCF complexes (where X and W = F, Cl, Br, and I) were well-characterized using various ab initio calculations. Geometrical optimization was first carried out for the investigated monomers at the MP2/aug-cc-pVTZ level of theory [66,67,68] for all atoms, except Br and I atoms. The aug-cc-pVTZ-PP basis set was utilized for the excepted atoms [66,67]. To envisage the electrophilic and nucleophilic regions over the molecular surfaces of the optimized molecules, the electrostatic potential (EP) analysis was carried out. In this perspective, molecular electrostatic potential (MEP) maps were built and then generated using 0.002 electron density envelopes according to the literature [69]. Further quantitative assessment was established by the evaluation of the surface electrostatic potential extrema in terms of *V*_s,min_ and *V*_s,max_ values, with the help of the Multiwfn 3.7 software [70], along the molecular surface of the inspected LB and LA, respectively.

The PoC approach was utilized to investigate the Lewis basicity and acidity roles on the tetrel-based interactions from an electrostatic perspective [18,47,50,71]. Using PoC approach, the effect of N∙∙∙ and C∙∙∙PoC distances were examined for the considered the NC-C_6_H_2_-X_3_/NCX (LB)∙∙∙ and W-C-F_3_∙∙∙/F-C-X_3_ (LA)∙∙∙PoC systems under the effect of PoC = ±0.50 au in distance range of 2.5–5.5 Å along the *x*-axis with a 0.1 Å step size. The molecular stabilization energies (*E*_stabilization_) were then evaluated and given from Equation (1) [63,72,73]:

(1) $$E_{\mathrm{stabilization}}=E_{\;\mathrm{molecule}\cdot \cdot \cdot \mathrm{PoC}}-E_{\mathrm{molecule}}$$

In order to thoroughly investigate the effect of Lewis basicity on interactions concerned with the carbon-bearing complexes, the NC-C_6_H_2_-X_3_ and NCX models were devoted to interacting with F-C-F_3_. Alternatively, the W-C-F_3_ and F-C-X_3_ molecules were adopted for interactions with NC-C_6_H_2_-F_3_ and NCF to outline the effect of Lewis acidity on tetrel bonding interactions. Geometrical optimization was performed for the designed Set I and II complexes. Using the optimized complexes, the interaction energies were evaluated using Equation (2). Principally, the Boys-Bernardi procedure was utilized to give a proper correction for the resulted interaction energies from the basis set superposition error (BSSE) [74].

(2) $$E_{\mathrm{int}\;}=E_{\mathrm{Set}\;I\;\mathrm{and}\;\mathrm{Set}\;\mathrm{II}\;\mathrm{complexes}}-\left(E_{\mathrm{LA}\;\mathrm{in}\;\mathrm{complex}}+E_{\mathrm{LB}\;\mathrm{in}\;\mathrm{complex}}\right)+E_{\mathrm{BSSE}}$$

where $E_{\mathrm{Set}-I\;\mathrm{and}\;\mathrm{Set}-\mathrm{II}\;\mathrm{complexes}}$, $E_{\mathrm{LA}\;\mathrm{in}\;\mathrm{complex}}$, and $E_{\mathrm{LB}\;\mathrm{in}\;\mathrm{complex}}$ represent energies of the complex, the deformed structures of LA, and LB, respectively. Energies of deformed monomers were considered based on their respective coordinates in the optimized Set I and II complexes.

To give qualitative and quantitative descriptions of the nature of the intermolecular interactions, quantum theory of atoms in molecules (QTAIM) [75] and noncovalent interaction (NCI) [64] analyses were carried out via Multiwfn 3.7 package [70]. The QTAIM diagrams and 2D/3D NCI plots were generated with the help of Visual Molecular Dynamics (VMD) software [76]. Gaussian 09 software was utilized for performing the executed calculations [77].

To investigate the physical behavior of the interactions embraced in the present study, symmetry-adapted perturbation theory (SAPT) analysis was performed at the SAPT2+(3)dMP2 level of truncation [78] using PSI4 code [79,80,81]. In the vein of SAPT, total SAPT2+(3)dMP2 energy was obtained as the sum of its physical nominees, including electrostatic (*E*_elst_), induction (*E*_ind_), dispersion (*E*_disp_), and exchange (*E*_exch_) terms, based on per Equations (3)–(7) [82].

(3) $$E_{\mathrm{int}}^{\mathrm{SAPT}2+\left(3\right)\mathrm{dMP}2}=E_{\mathrm{elst}}+E_{\mathrm{exch}}+E_{\mathrm{ind}}+E_{\mathrm{disp}}$$

(4) $$E_{\mathrm{elst}}=E_{\mathrm{elst}}^{\left(10\right)}+E_{\mathrm{elst}}^{\left(12\right)}+E_{\mathrm{elst}}^{\left(13\right)}$$

(5) $$E_{\mathrm{exch}}=E_{\mathrm{exch}}^{\left(10\right)}+E_{\mathrm{exch}}^{\left(11\right)}+E_{\mathrm{exch}}^{\left(12\right)}$$

(6) $$E_{\mathrm{ind}}=E_{\mathrm{ind},\mathrm{resp}}^{\left(20\right)}+E_{\mathrm{exch}-\mathrm{ind},\mathrm{resp}}^{\left(20\right)}+E_{\mathrm{ind}}^{\left(22\right)}+E_{\mathrm{exch}-\mathrm{ind}}^{\left(22\right)}+\;\delta E_{\mathrm{HF}}^{\left(2\right)}+\delta E_{\mathrm{MP}2\;}^{\left(2\right)}$$

(7) $$E_{\mathrm{disp}}=E_{\mathrm{disp}}^{\left(20\right)}+E_{\mathrm{exch}-\mathrm{disp}}^{\left(20\right)}+E_{\mathrm{disp}}^{\left(21\right)}+E_{\mathrm{disp}\;}^{\left(22\right)}\left(\mathrm{SDQ}\right)+E_{\mathrm{disp}}^{\left(22\right)}T+E_{\mathrm{disp}}^{\left(30\right)}$$

## 4. Conclusions

Ab initio calculations were performed to investigate the effects of Lewis basicity and acidity on the tetrel bonding interactions using F-C-F_3_∙∙∙NC-C_6_H_2_-X_3_/NCX and W-C-F_3_/F-C-X_3_∙∙∙NC-C_6_H_2_-F_3_/NCF complexes (i.e., Set I and II complexes), respectively. Regarding the Lewis basicity effect, the MP2 energetic quantities of Set I complexes further confirmed the favorability of the F-C-F_3_∙∙∙NC-C_6_H_2_-X_3_ complexes with favorable *E*_int_ (i.e., more negative) over their F-C-F_3_∙∙∙NCX counterparts. Additionally, *E*_int_ of Set I complexes increased along with decreasing the electron-withdrawing power of the X_3_/X halogen(s) in the following order F < Cl < Br < I. Regarding the Lewis acidity effects, *V*_s,max_ quantities exhibited higher values in the case of W-C-F_3_ molecules compared with F-C-X_3_. Nevertheless, higher negative *E*_int_ were ascribed to the F-C-X_3_ bearing complexes compared with the W-C-F_3_ bearing ones. QTAIM diagrams and NCI plots validated the prominent contributions of the X_3_ halogen substituents. Moreover, SAPT observations identified *E*_disp_ as the most dominant energetic aspect that contributed to the total strength of all the studied complexes. These observations provide informative characteristics of carbon-bearing complexes that may be fundamental in future studies related to crystal engineering and materials science.

## Figures and Tables

**Table 1:** Molecular stabilization (*E*_stabilization_) and destabilization (*E*_destabilization_) energies of (a) NC-C_6_H_2_-X_3_∙∙∙, (b) NCX∙∙∙, (c) W-C-F_3_∙∙∙, and (d) F-C-X_3_∙∙∙PoC systems (i.e., X and W = F, Cl, Br, and I) under the effect of PoC = ±0.50 au at N/C∙∙∙PoC distance of 2.5 Å.

Molecular Energies (i.e., *E*_destabilization_ and *E*_stabilization,_ in kcal/mol)				
Lewis base∙∙∙PoC systems				
System	(a) NC-C_6_H_2_-X_3_∙∙∙PoC		(b) NCX∙∙∙PoC	
	−0.50 au	+0.50 au	−0.50 au	+0.50 au
F	7.41	−13.06	7.13	−11.20
Cl	7.44	−13.39	7.62	−11.40
Br	7.59	−13.64	7.85	−12.90
I	7.72	−13.92	8.15	−13.51
Lewis acid∙∙∙PoC systems				
System	(c) W-C-F_3_∙∙∙PoC		(d) F-C-X_3_∙∙∙PoC	
	−0.50 au	+0.50 au	−0.50 au	+0.50 au
F	−5.58	0.30	−5.58	0.30
Cl	−4.98	−1.34	−7.88	−5.81
Br	−4.96	−1.75	−9.11	−8.18
I	−4.62	−2.66	−12.12	−10.41

**Table 2:** Interaction energies (*E*_int_, in kcal/mol) for (a) Set I and (b) Set II complexes were evaluated at the MP2/aug-cc-pVTZ(PP) level of theory.

W/X	Complexation Parameters		Complexation Parameters	
	Distance (Å)	*E*_MP2/aug−cc−pVTZ(PP)_ (kcal/mol)	Distance (Å)	*E*_MP2/aug−cc−pVTZ(PP)_ (kcal/mol)
(a) Set I complexes				
	F-C-F_3_∙∙∙NC-C_6_H_2_-X_3_		F-C-F_3_∙∙∙NCX	
F	3.33	−1.20	3.35	−1.05
Cl	3.33	−1.21	3.34	−1.13
Br	3.31	−1.21	3.31	−1.13
I	3.32	−1.23	3.30	−1.16
(b) Set II complexes				
	W-C-F_3_∙∙∙NC-C_6_H_2_-F_3_		W-C-F_3_∙∙∙NCF	
F	3.33	−1.20	3.35	−1.05
Cl	3.37	−1.07	3.38	−0.91
Br	3.35	−1.07	3.37	−0.91
I	3.36	−0.97	3.38	−0.82
	F-C-X_3_∙∙∙NC-C_6_H_2_-F_3_		F-C-X_3_∙∙∙NCF	
F	3.33	−1.20	3.35	–1.05
Cl	3.55	–1.55	3.60	–1.25
Br	3.72 ^a^	–1.66 ^a^	3.57	–1.25
I	3.75 ^a^	–2.08 ^a^	3.58	–1.46

^a^ *E*_int_ was computed from the potential energy surface (PES) scan depicted in Figure S2.

**Table 3:** Topological parameters, comprising *ρ*_b_, ∇^2^*ρ*_b_, and H_b_ (in au), for (a) Set I and (b) Set II complexes.

W/X	*ρ*_b_ (au)	∇^2^*ρ*_b_ (au)	H_b_ (au)	*ρ*_b_ (au)	∇^2^*ρ*_b_ (au)	H_b_ (au)
(a) Set I complexes						
	F-C-F_3_∙∙∙NC-C_6_H_2_-X_3_			F-C-F_3_∙∙∙NCX		
F	0.005043	0.020899	0.001060	0.004768	0.019982	0.001032
Cl	0.005062	0.020980	0.001064	0.004907	0.020402	0.001041
Br	0.005260	0.021898	0.001106	0.005265	0.021980	0.001110
I	0.005130	0.021289	0.001078	0.005317	0.022116	0.001111
(b) Set II complexes						
	W-C-F_3_∙∙∙NC-C_6_H_2_-F_3_			W-C-F_3_∙∙∙NCF		
F	0.005033	0.020836	0.001056	0.004768	0.019982	0.001032
Cl	0.004860	0.019906	0.001005	0.004558	0.018900	0.000974
Br	0.004977	0.020428	0.001028	0.004660	0.019332	0.000993
I	0.005258	0.022034	0.000912	0.004624	0.019122	0.000981
	F-C-X_3_∙∙∙NC-C_6_H_2_-F_3_			F-C-X_3_∙∙∙NCF		
F	0.005033	0.020836	0.001056	0.004768	0.019982	0.001032
Cl	0.005739	0.017131	0.000847	0.005088	0.018951	0.001156
Br	0.005030 ^a^	0.016791 ^a^	0.000928 ^a^	0.006197	0.021593	0.001184
I	0.005591 ^a^	0.020600 ^a^	0.001220 ^a^	0.006986	0.021434	0.001017

^a^ The QTAIM parameters were recorded at the most favorable parameters based on the PES scan depicted in Figure S2.

## Data Availability

Data is contained within the article.

## Supplementary Materials

The supporting information can be downloaded at: https://www.mdpi.com/article/10.3390/ijms232113023/s1.
