# Supplementary material for: Effects of Lewis Basicity and Acidity on σ-Hole Interactions in Carbon-Bearing Complexes: A Comparative Ab Initio Study
Source: Int J Mol Sci. 2022 Oct 27;23(21):13023. doi: 10.3390/ijms232113023 (PMC9658749; doi:10.3390/ijms232113023)
Supplement: Supplementary file 1 [file ijms-23-13023-s001.zip › ijms-1954606-supplementary.pdf]

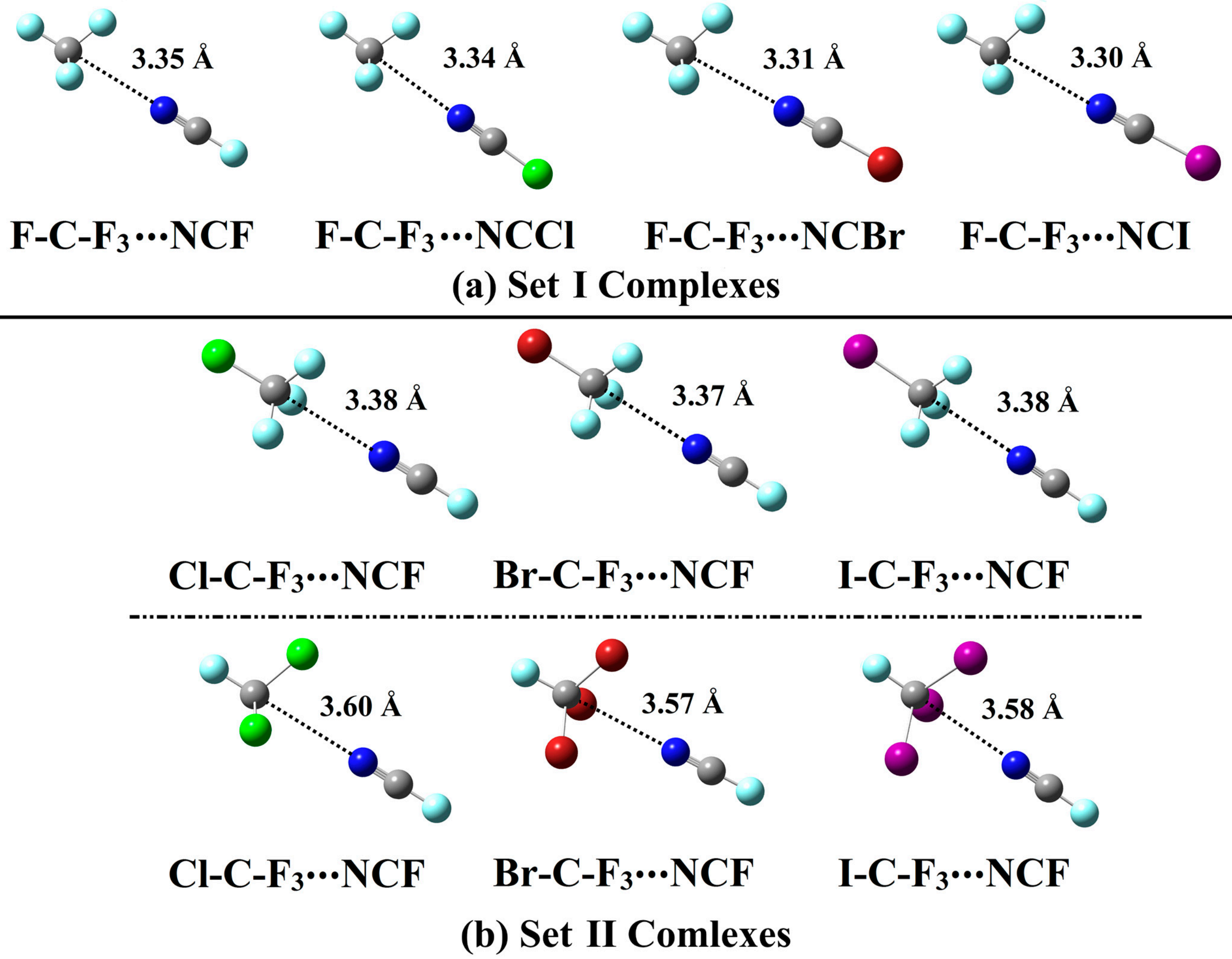

Figure S1. (**a**) Set I and (**b**) Set II complexes elucidating the Lewis basicity and acidity effects, respectively. The C···N distances are evaluated in Å.

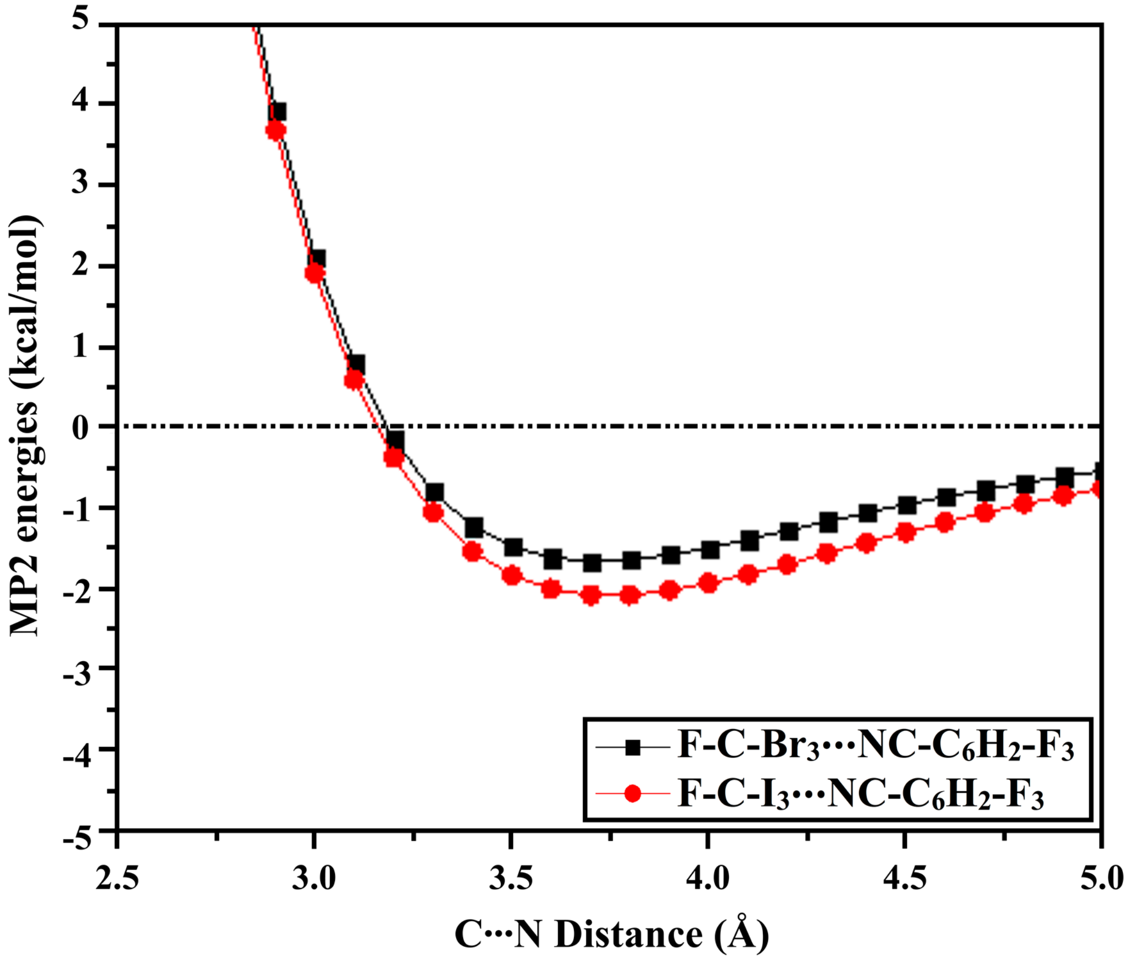

Figure S2. MP2 energies for the F-C-Br_3/ F-C-I_3 ···NC-C_6H_2-F_3 complexes. The C···N distances are evaluated in Å.

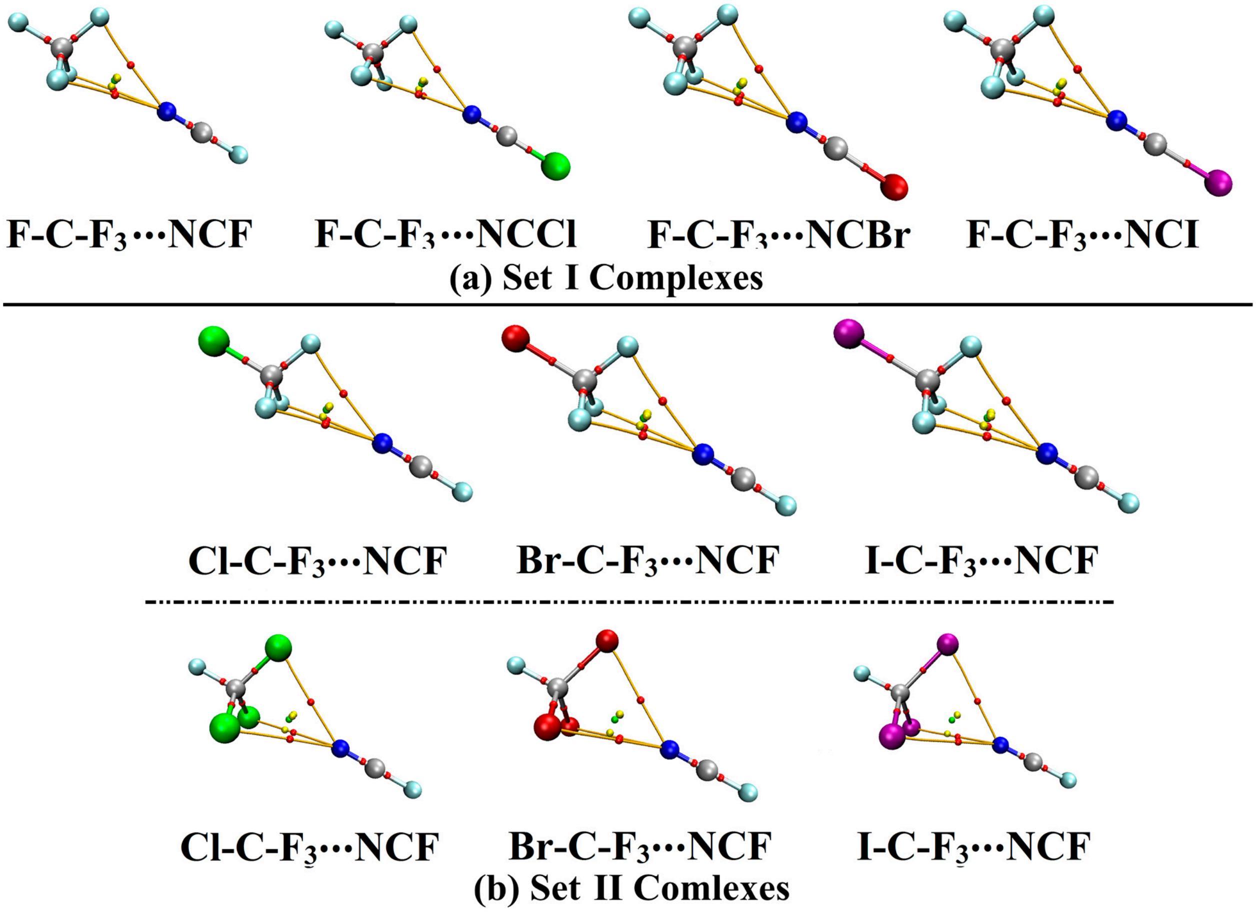

Figure S3. QTAIM diagrams of (**a**) Set I and (**b**) Set II complexes. Red dots point out the BCPs locations within the interacting species.

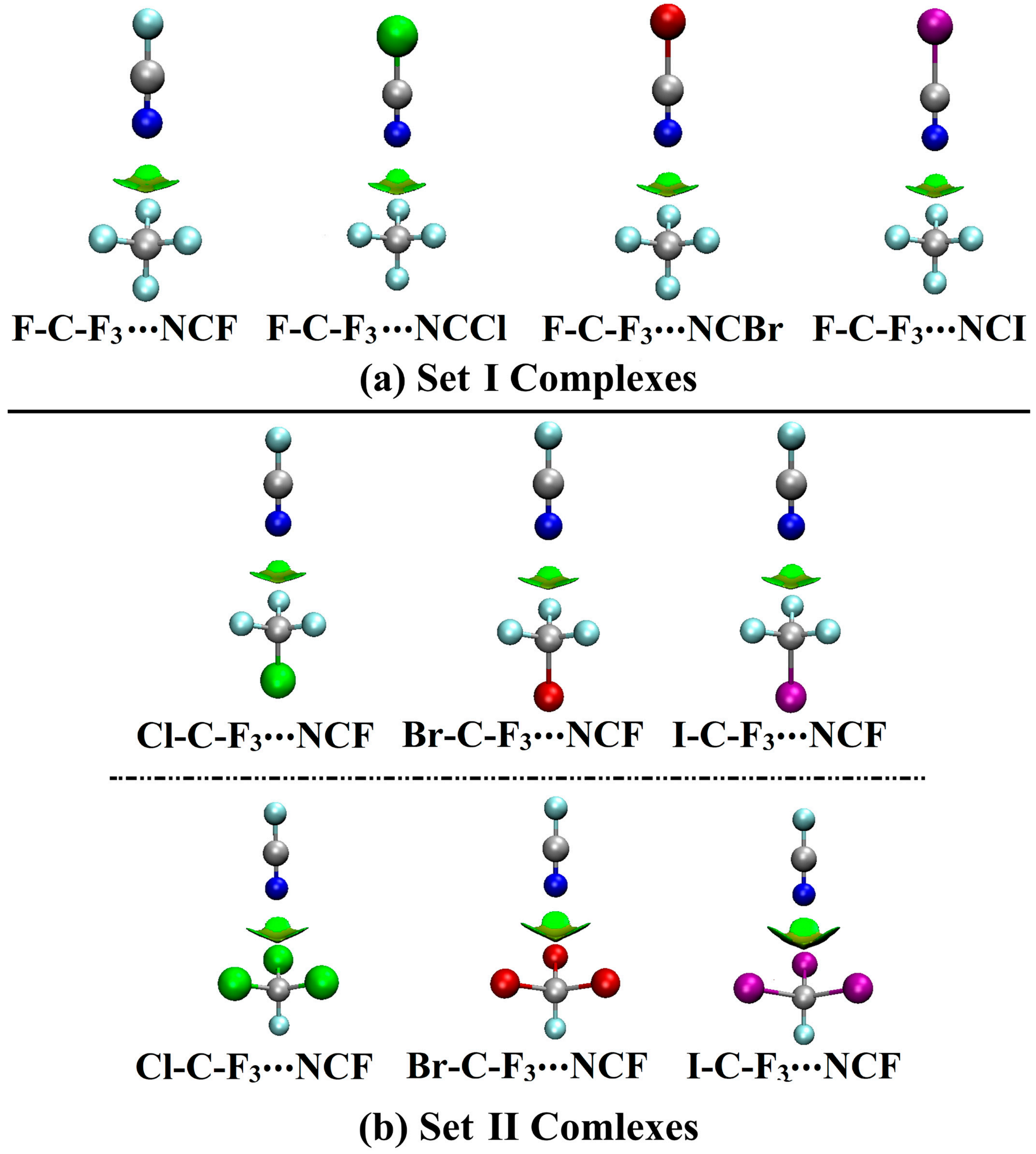

Figure S4. 3D NCI plots of the (**a**) Set I complexes and (**b**) Set II complexes. The color scope extended based on the sign(λ2)ϱ from −0.035 to 0.020 au (blue to red), where ϱ represents the electron density and λ2 refers to the second eigenvalue of the Hessian matrix based on the sign(λ2)ρ.

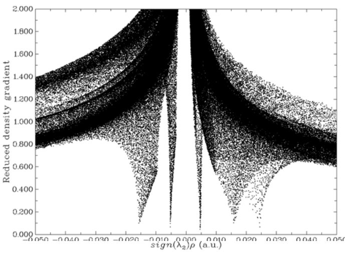

F-C-F_3···NC-C_6H_2-F_3

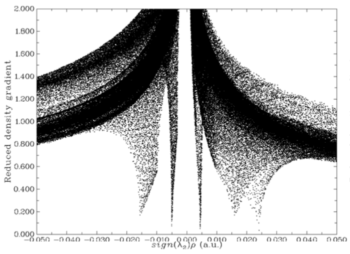

F-C-F_3···NC-C_6H_2-Cl_3

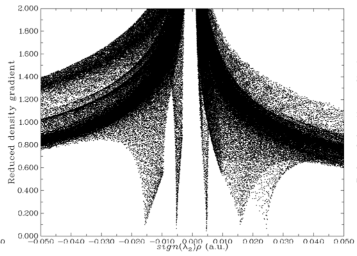

F-C-F_3···NC-C_6H_2-Br_3

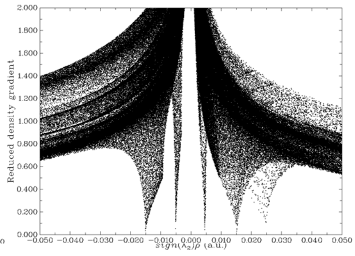

F-C-F_3···NC-C_6H_2-I

(a) Set-I Complexes

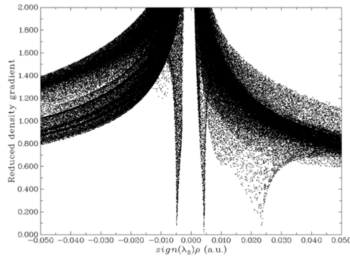

Cl-C-F_3···NC-C_6H_2-F_3

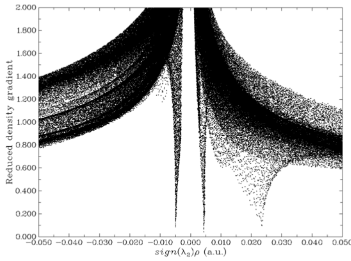

Br-C-F_3···NC-C_6H_2-F_3

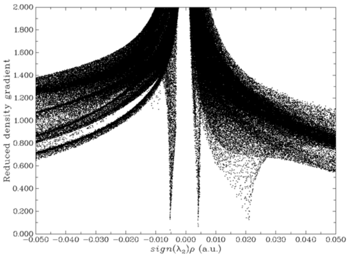

I-C-F_3···NC-C_6H_2-F_3

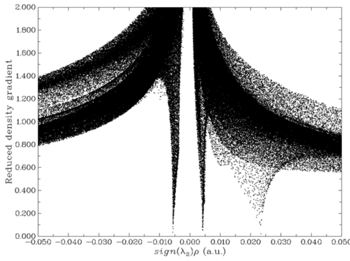

F-C-Cl_3···NC-C_6H_2-F_3

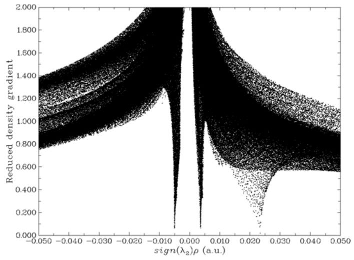

F-C-Br_3···NC-C_6H_2-F_3

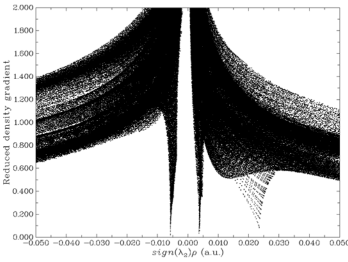

F-C-I_3···NC-C_6H_2-F_3

(b) Set-II Comlexes

Figure S5. 2D noncovalent interaction (NCI) reduced density gradient (RDG) plots for the (**a**) Set I complexes and (**b**) Set II complexes.

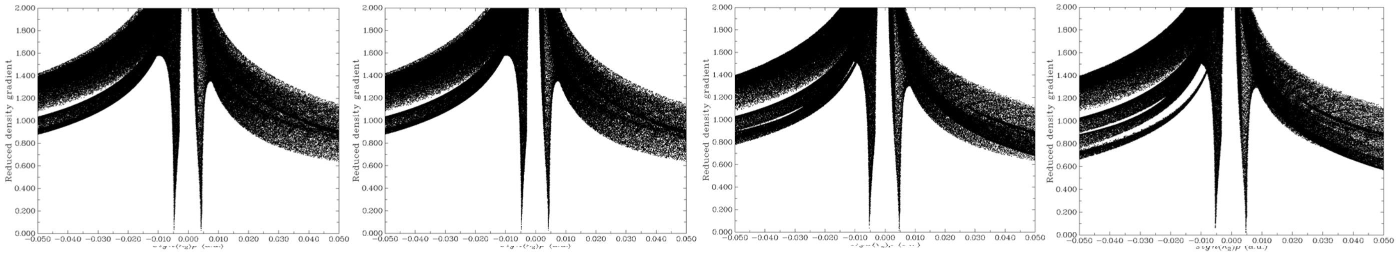

F-C-F_3···NCF F· F-C-F_3···NCCl F-C-F_3···NCBr F-C-F_3···NCI

(a) Set-I Complexes

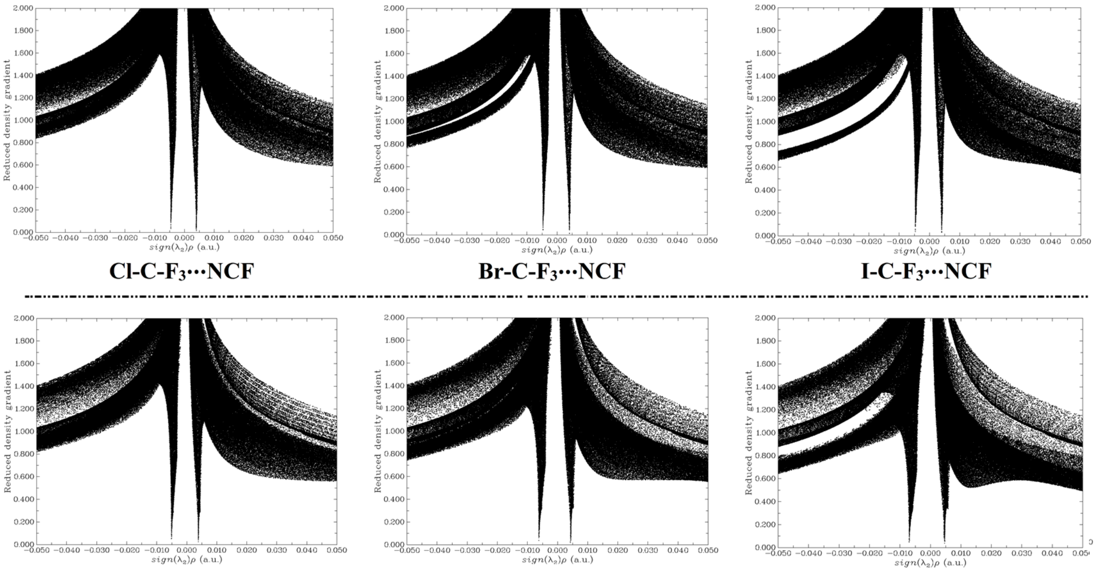

Cl-C-F_3···NCF Br-C-F_3···NCF I-C-F_3···NCF

Cl-C-F_3···NCF Br-C-F_3···NCF I-C-F_3···NCF

(b) Set-II Comlexes

Figure S6. 2D noncovalent interaction (NCI) reduced density gradient (RDG) plots for the (**a**) Set I complexes and (**b**) Set II complexes.

Table S1. E_{elst}, E_{ind}, E_{disp}, and E_{exch}, along with total $E_{SAPT2+(3)dMP2}$ (in kcal/mol) of the F-C-F_3···NC-C_6H_2-X_3/NCX and W-C-F_3/F-C-X_3···NC-C_6H_2-F_3/NCF complexes.

W/X	Complexation parameters					Complexation parameters				
	E_{elst}	E_{ind}	E_{disp}	E_{exch}	$E_{SAPT2+(3)dMP2}$[a]	E_{elst}	E_{ind}	E_{disp}	E_{exch}	$E_{SAPT2+(3)dMP2}$[a]
	F-C-F_3···NC-C_6H_2-X_3					F-C-F_3···NCX				
F	-1.49	-0.05	-1.93	2.20	−1.28	-1.26	-0.03	-1.73	1.91	−1.10
Cl	-1.51	-0.05	-1.96	2.22	−1.30	-1.37	-0.03	-1.83	1.91	−1.18
Br	-1.59	-0.06	-2.03	2.38	−1.30	-1.52	-0.03	-1.97	2.05	−1.19
I	-1.55	-0.06	-2.00	2.28	−1.33	-1.57	-0.04	-2.01	2.34	−1.22
	W-C-F_3···NC-C_6H_2-F_3					W-C-F_3···NCF				
F	−1.49	−0.05	−1.93	2.20	−1.28	−1.26	−0.03	−1.73	1.91	−1.10
Cl	−1.14	−0.06	−1.97	2.08	−1.10	−0.95	−0.03	−1.74	1.81	−0.92
Br	−1.15	−0.07	−2.07	2.21	−1.08	−0.95	−0.04	−1.81	1.90	−0.90
I	−1.00	−0.08	−2.12	2.25	−0.95	−0.80	−0.05	−1.84	1.90	−0.79
	F-C-X_3···NC-C_6H_2-F_3					F-C-X_3···NCF				
F	−1.49	−0.05	−1.93	2.20	−1.28	−1.26	−0.03	−1.73	1.91	−1.10
Cl	−1.35	−0.28	−3.52	3.88	−1.27	−1.09	−0.20	−3.00	3.23	−1.05
Br	−0.99[b]	−0.40[b]	−3.64[b]	3.59[b]	−1.43[b]	−1.59	−0.42	−4.01	5.03	−0.99
I	−1.73[b]	−0.71[b]	−4.79[b]	5.44[b]	−1.79[b]	−2.66	−0.78	−5.24	7.53	−1.15

[a] $E_{SAPT2+(3)dMP2} = E_{elst} + E_{ind} + E_{disp} + E_{exch}$.

[b]The SAPT results were recorded based on the PES scan depicted in Figure S2.

For Set I Complexes:

$F\text{-}C\text{-}F_3\cdots NC\text{-}C_6H_2\text{-}F_3$

Molecule Name
18 17
SMALL
NO_CHARGES

@<TRIPOS>ATOM
1 C1 -1.2167 -0.3117 0.0109 C
2 F2 -0.7776 0.3101 -1.0665 F
3 F3 -0.7685 -1.5523 0.0273 F
4 F4 -0.7726 0.3234 1.0886 F
5 F5 -2.5358 -0.3105 0.0238 F
6 N6 -2.3306 -1.9038 -2.6864 N
7 C7 -2.7211 -2.4609 -3.6432 C
8 C8 -3.1957 -3.1387 -4.8082 C
9 C9 -2.4348 -4.1634 -5.3716 C
10 C10 -4.4167 -2.7713 -5.3743 C
11 C11 -2.8987 -4.8237 -6.5078 C
12 H12 -1.4897 -4.4426 -4.9274 H
13 C13 -4.8784 -3.4332 -6.5105 C
14 H14 -5.0008 -1.9764 -4.9322 H
15 C15 -4.1289 -4.4717 -7.0990 C
16 I16 -6.7093 -2.7918 -7.2551 I
17 I17 -4.8166 -5.4540 -8.7870 I
18 I18 -1.6729 -6.3294 -7.2482 I
@<TRIPOS>BOND
1 1 2 1
2 1 3 1
3 1 4 1
4 1 5 1
5 6 7 3
6 7 8 Ar
7 8 9 Ar
8 8 10 Ar
9 9 11 Ar
10 9 12 1
11 10 13 Ar
12 10 14 1
13 11 15 Ar
14 11 18 1

15 13 15 Ar
16 13 16 1
17 15 17 1

$F\text{-}C\text{-}F_3\cdots NC\text{-}C_6H_2\text{-}Cl_3$

```
 Molecule Name
18 17
SMALL
NO_CHARGES


@<TRIPOS>ATOM
1 C1      -1.2101   -0.3158    0.0261 C
2 F2      -0.7671    0.3111   -1.0468 F
3 F3      -0.7654   -1.5578    0.0359 F
4 F4      -0.7667    0.3115    1.1084 F
5 F5      -2.5293   -0.3112    0.0361 F
6 N6      -2.3248   -1.8927   -2.6881 N
7 C7      -2.7154   -2.4451   -3.6474 C
8 C8      -3.1905   -3.1175   -4.8159 C
9 C9      -2.4288   -4.1427   -5.3804 C
10 C10    -4.4112   -2.7420   -5.3804 C
11 C11    -2.8964   -4.7925   -6.5166 C
12 H12    -1.4837   -4.4350   -4.9451 H
13 C13    -4.8674   -3.3999   -6.5166 C
14 H14    -5.0024   -1.9488   -4.9452 H
15 C15    -4.1200   -4.4332   -7.1022 C
16 Cl16   -6.3723   -2.9238   -7.1973 Cl
17 Cl17   -4.6882   -5.2375   -8.5000 Cl
18 Cl18   -1.9450   -6.0520   -7.1971 Cl
@<TRIPOS>BOND
1 1 2 1
2 1 3 1
3 1 4 1
4 1 5 1
5 6 7 3
6 7 8 Ar
7 8 9 Ar
8 8 10 Ar
9 9 11 Ar
10 9 12 1
11 10 13 Ar
12 10 14 1
13 11 15 Ar
14 11 18 1
15 13 15 Ar
16 13 16 1
```

17 15 17 1

$F\text{-}C\text{-}F_3\cdots NC\text{-}C_6H_2\text{-}Br_3$

Molecule Name
18 17
SMALL
NO_CHARGES

@<TRIPOS>ATOM
1 C1 -1.2280 -0.3305 -0.0099 C
2 F2 -0.7938 0.2992 -1.0847 F
3 F3 -0.7723 -1.5685 0.0000 F
4 F4 -0.7865 0.3019 1.0704 F
5 F5 -2.5471 -0.3373 0.0045 F
6 N6 -2.3306 -1.9089 -2.7037 N
7 C7 -2.7201 -2.4642 -3.6617 C
8 C8 -3.1943 -3.1400 -4.8281 C
9 C9 -2.4275 -4.1580 -5.3974 C
10 C10 -4.4202 -2.7761 -5.3879 C
11 C11 -2.8939 -4.8124 -6.5326 C
12 H12 -1.4784 -4.4397 -4.9637 H
13 C13 -4.8768 -3.4372 -6.5232 C
14 H14 -5.0138 -1.9879 -4.9469 H
15 C15 -4.1239 -4.4647 -7.1147 C
16 Br16 -6.5234 -2.9091 -7.2290 Br
17 Br17 -4.7408 -5.3439 -8.6321 Br
18 Br18 -1.8267 -6.1662 -7.2514 Br
@<TRIPOS>BOND
1 1 2 1
2 1 3 1
3 1 4 1
4 1 5 1
5 6 7 3
6 7 8 Ar
7 8 9 Ar
8 8 10 Ar
9 9 11 Ar
10 9 12 1
11 10 13 Ar
12 10 14 1
13 11 15 Ar
14 11 18 1
15 13 15 Ar
16 13 16 1

17 15 17 1

$F\text{-}C\text{-}F_3\cdots NC\text{-}C_6H_2\text{-}I_3$

Molecule Name
18 17
SMALL
NO_CHARGES

@<TRIPOS>ATOM
1 C1 -1.2167 -0.3117 0.0109 C
2 F2 -0.7776 0.3101 -1.0665 F
3 F3 -0.7685 -1.5523 0.0273 F
4 F4 -0.7726 0.3234 1.0886 F
5 F5 -2.5358 -0.3105 0.0238 F
6 N6 -2.3306 -1.9038 -2.6864 N
7 C7 -2.7211 -2.4609 -3.6432 C
8 C8 -3.1957 -3.1387 -4.8082 C
9 C9 -2.4348 -4.1634 -5.3716 C
10 C10 -4.4167 -2.7713 -5.3743 C
11 C11 -2.8987 -4.8237 -6.5078 C
12 H12 -1.4897 -4.4426 -4.9274 H
13 C13 -4.8784 -3.4332 -6.5105 C
14 H14 -5.0008 -1.9764 -4.9322 H
15 C15 -4.1289 -4.4717 -7.0990 C
16 I16 -6.7093 -2.7918 -7.2551 I
17 I17 -4.8166 -5.4540 -8.7870 I
18 I18 -1.6729 -6.3294 -7.2482 I
@<TRIPOS>BOND
1 1 2 1
2 1 3 1
3 1 4 1
4 1 5 1
5 6 7 3
6 7 8 Ar
7 8 9 Ar
8 8 10 Ar
9 9 11 Ar
10 9 12 1
11 10 13 Ar
12 10 14 1
13 11 15 Ar
14 11 18 1
15 13 15 Ar
16 13 16 1

17 15 17 1

F-C-F_3···NCF

```
  Molecule Name
8 6
SMALL
NO_CHARGES


@<TRIPOS>ATOM
1 C1    -1.2066   -0.3112    0.0383 C
2 F2    -0.7627    0.3165   -1.0341 F
3 F3    -0.7628   -1.5538    0.0457 F
4 F4    -0.7645    0.3140    1.1213 F
5 F5    -2.5260   -0.3069    0.0457 F
6 N6    -2.3221   -1.8887   -2.6940 N
7 C7    -2.7125   -2.4409   -3.6505 C
8 F8    -3.1351   -3.0385   -4.6855 F
@<TRIPOS>BOND
1 1 2 1
2 1 3 1
3 1 4 1
4 1 5 1
5 6 7 3
6 7 8 1
```

F-C-F_3···NCCl

```
  Molecule Name
8 6
SMALL
NO_CHARGES


@<TRIPOS>ATOM
1 C1     -1.2092    -0.3149     0.0319 C
2 F2     -0.7651     0.3132    -1.0400 F
3 F3     -0.7651    -1.5572     0.0399 F
4 F4     -0.7668     0.3107     1.1153 F
5 F5     -2.5284    -0.3102     0.0399 F
6 N6     -2.3224    -1.8892    -2.6948 N
7 C7     -2.7141    -2.4431    -3.6543 C
8 Cl8    -3.2578    -3.2119    -4.9858 Cl
@<TRIPOS>BOND
1 1 2 1
2 1 3 1
3 1 4 1
4 1 5 1
5 6 7 3
6 7 8 1
```

F-C-F_3···NCBr

Molecule Name
8 6
SMALL
NO_CHARGES

@<TRIPOS>ATOM

1 C1	-1.2138	-0.3214	0.0208	C
2 F2	-0.7694	0.3071	-1.0506	F
3 F3	-0.7694	-1.5635	0.0293	F
4 F4	-0.7714	0.3042	1.1046	F
5 F5	-2.5330	-0.3164	0.0293	F
6 N6	-2.3155	-1.8793	-2.6780	N
7 C7	-2.7073	-2.4335	-3.6378	C
8 Br8	-3.2992	-3.2707	-5.0878	Br

@<TRIPOS>BOND
1 1 2 1
2 1 3 1
3 1 4 1
4 1 5 1
5 6 7 3
6 7 8 1

F-C-F_3···NCI

```
  Molecule Name
8 6
SMALL
NO_CHARGES


@<TRIPOS>ATOM
1 C1      -1.2134     -0.3208      0.0217 C
2 F2      -0.7689      0.3078     -1.0495 F
3 F3      -0.7689     -1.5628      0.0305 F
4 F4      -0.7708      0.3050      1.1056 F
5 F5      -2.5325     -0.3157      0.0305 F
6 N6      -2.3143     -1.8778     -2.6751 N
7 C7      -2.7065     -2.4325     -3.6358 C
8 I8     -3.3670     -3.3662     -5.2534 I
@<TRIPOS>BOND
1 1 2 1
2 1 3 1
3 1 4 1
4 1 5 1
5 6 7 3
6 7 8 1
```

For Set II Complexes (W-Effect)

$Cl\text{-}C\text{-}F_3 \cdots NC\text{-}C_6H_2\text{-}F_3$

```
 Molecule Name
18 17
SMALL
NO_CHARGES


@<TRIPOS>ATOM
1 C1 5.3919 1.7202 8.7388 C
2 F2 5.0848 0.7658 9.6044 F
3 Cl3 6.5818 2.8085 9.4453 Cl
4 F4 4.2940 2.3935 8.4293 F
5 F5 5.8843 1.1666 7.6409 F
6 C6 -0.5329 -3.6603 5.1880 C
7 C7 0.7193 -4.1376 5.5647 C
8 C8 1.6681 -3.3022 6.1268 C
9 C9 1.3469 -1.9539 6.3135 C
10 C10 0.0960 -1.4495 5.9436 C
11 C11 -0.8280 -2.3142 5.3844 C
12 H12 2.6323 -3.6989 6.4101 H
13 H13 -0.1593 -0.4093 6.0848 H
14 F14 -2.0312 -1.8714 5.0188 F
15 F15 -1.4316 -4.4761 4.6499 F
16 F16 0.9877 -5.4291 5.3706 F
17 C17 2.3144 -1.0755 6.8929 C
18 N18 3.1087 -0.3541 7.3686 N
@<TRIPOS>BOND
1 1 2 1
2 1 3 1
3 1 4 1
4 1 5 1
5 6 7 Ar
6 6 11 Ar
7 6 15 1
8 7 8 2
9 7 16 1
10 8 9 Ar
11 8 12 1
12 9 10 Ar
13 9 17 Ar
14 10 11 2
```

15 10 13 1
16 11 14 1
17 17 18 3

$Br\text{-}C\text{-}F_3\cdots NC\text{-}C_6H_2\text{-}F_3$

Molecule Name
18 17
SMALL
NO_CHARGES

@<TRIPOS>ATOM
1 C1 4.1438 2.9793 3.7664 C
2 F2 4.9925 2.0347 3.3890 F
3 F3 2.9816 2.4216 4.0712 F
4 F4 3.9679 3.8293 2.7658 F
5 Br5 4.8407 3.9077 5.2942 Br
6 N6 2.9259 1.3528 1.0922 N
7 C7 2.5004 0.7841 0.1576 C
8 C8 1.9824 0.0915 -0.9805 C
9 C9 2.7232 -0.9516 -1.5454 C
10 C10 0.7439 0.4691 -1.5091 C
11 C11 2.2063 -1.6107 -2.6465 C
12 H12 3.6806 -1.2489 -1.1425 H
13 C13 0.2582 -0.2124 -2.6108 C
14 H14 0.1661 1.2738 -1.0781 H
15 C15 0.9761 -1.2541 -3.1916 C
16 F16 -0.9184 0.1180 -3.1437 F
17 F17 0.4950 -1.8974 -4.2486 F
18 F18 2.8825 -2.6103 -3.2133 F
@<TRIPOS>BOND
1 1 2 1
2 1 3 1
3 1 4 1
4 1 5 1
5 6 7 3
6 7 8 Ar
7 8 9 Ar
8 8 10 Ar
9 9 11 2
10 9 12 1
11 10 13 2
12 10 14 1
13 11 15 Ar
14 11 18 1
15 13 15 Ar
16 13 16 1

17 15 17 1

I-C-F_3···NC-C_6H_2-F_3

Molecule Name
18 17
SMALL
NO_CHARGES

@<TRIPOS>ATOM
1 C1 2.9819 2.1457 2.7043 C
2 F2 3.8524 1.2186 2.3239 F
3 F3 1.8110 1.5655 2.9371 F
4 F4 2.8368 3.0270 1.7224 F
5 I5 3.6797 3.1258 4.4726 I
6 N6 1.8859 0.6036 -0.0764 N
7 C7 1.5045 0.0645 -1.0469 C
8 C8 1.0402 -0.5922 -2.2286 C
9 C9 1.8136 -1.6087 -2.7983 C
10 C10 -0.1793 -0.2068 -2.7945 C
11 C11 1.3486 -2.2332 -3.9419 C
12 H12 2.7566 -1.9118 -2.3668 H
13 C13 -0.6129 -0.8534 -3.9381 C
14 H14 -0.7821 0.5775 -2.3600 H
15 C15 0.1383 -1.8681 -4.5245 C
16 F16 -1.7700 -0.5145 -4.5072 F
17 F17 -0.2929 -2.4781 -5.6221 F
18 F18 2.0572 -3.2067 -4.5146 F
@<TRIPOS>BOND
1 1 2 1
2 1 3 1
3 1 4 1
4 1 5 1
5 6 7 3
6 7 8 Ar
7 8 9 Ar
8 8 10 Ar
9 9 11 2
10 9 12 1
11 10 13 2
12 10 14 1
13 11 15 Ar
14 11 18 1
15 13 15 Ar
16 13 16 1

17 15 17 1

Cl-C-F_3···NCF

```
 Molecule Name
8 6
SMALL
NO_CHARGES


@<TRIPOS>ATOM
1 C1    -0.9415    1.7501    -0.1572 C
2 F2    -1.4191    2.9769    -0.3053 F
3 F3    -1.4300    0.9780    -1.1165 F
4 F4    -1.3279    1.2754     1.0178 F
5 Cl5    0.8155    1.7761    -0.2449 Cl
6 N6    -4.3260    1.7000     0.0117 N
7 C7    -5.4959    1.6827     0.0701 C
8 F8    -6.7621    1.6644     0.1333 F
@<TRIPOS>BOND
1 1 2 1
2 1 3 1
3 1 4 1
4 1 5 1
5 6 7 3
6 7 8 1
```

Br-C-F_3···NCF

```
 Molecule Name
8 6
SMALL
NO_CHARGES


@<TRIPOS>ATOM
1 C1    -0.7555     1.6511    -0.1011 C
2 F2    -1.1883     2.8932    -0.2606 F
3 F3    -1.2483     0.8937    -1.0702 F
4 F4    -1.1817     1.1913     1.0662 F
5 Br5     1.1608     1.6158    -0.1559 Br
6 N6    -4.1309     1.7133    -0.0045 N
7 C7    -5.3017     1.7349     0.0290 C
8 F8    -6.5688     1.7575     0.0653 F
@<TRIPOS>BOND
1 1 2 1
2 1 3 1
3 1 4 1
4 1 5 1
5 6 7 3
6 7 8 1
```

I-C-F_3···NCF

```
 Molecule Name
8 6
SMALL
NO_CHARGES


@<TRIPOS>ATOM
1 C1   -3.1508   1.3673   -0.0356 C
2 F2   -3.6807   2.5732   -0.2020 F
3 F3   -3.5729   0.5757   -1.0143 F
4 F4   -3.5674   0.8732    1.1242 F
5 I5   -1.0168   1.4918   -0.0584 I
6 N6   -6.5324   1.1697    0.0012 N
7 C7   -7.7019   1.1015    0.0139 C
8 F8   -8.9677   1.0278    0.0277 F
@<TRIPOS>BOND
1 1 2 1
2 1 3 1
3 1 4 1
4 1 5 1
5 6 7 3
6 7 8 1
```

For Set-II Complexes (X_3-Effect)

F-C-Cl_3···NC-C_6H_2-F_3

```
 Molecule Name
18 17
SMALL
NO_CHARGES


@<TRIPOS>ATOM
1 C1     1.8847     1.9057     3.4772 C
2 F2     2.2229     2.5755     4.5960 F
3 Cl3     2.5452     2.7750     2.0975 Cl
4 Cl4     0.1293     1.8374     3.3885 Cl
5 Cl5     2.5675     0.2878     3.5793 Cl
6 N6     0.9914     0.1362     0.5240 N
7 C7     0.6965    -0.4477    -0.4513 C
8 C8     0.3380    -1.1577    -1.6388 C
9 C9    -0.4159    -2.3312    -1.5362 C
10 C10     0.7474    -0.6666    -2.8827 C
11 C11    -0.7537    -3.0053    -2.6962 C
12 H12    -0.7357    -2.7167    -0.5789 H
13 C13     0.3912    -1.3669    -4.0215 C
14 H14     1.3299     0.2392    -2.9699 H
15 C15    -0.3586    -2.5373    -3.9462 C
16 F16     0.7625    -0.9308    -5.2254 F
17 F17    -0.6916    -3.1968    -5.0493 F
18 F18    -1.4714    -4.1274    -2.6397 F
@<TRIPOS>BOND
1 1 2 1
2 1 3 1
3 1 4 1
4 1 5 1
5 6 7 3
6 7 8 Ar
7 8 9 Ar
8 8 10 Ar
9 9 11 2
10 9 12 1
11 10 13 2
12 10 14 1
13 11 15 Ar
14 11 18 1
15 13 15 Ar
```

16 13 16 1
17 15 17 1

F-C-Br_3···NC-C_6H_2-F_3

Molecule Name
18 17
SMALL
NO_CHARGES

@<TRIPOS>ATOM

1 C1	-0.0000	0.0000	0.0000	C
2 F2	-1.3447	-0.0000	-0.0000	F
3 Br3	0.6011	0.0000	1.8210	Br
4 Br4	0.6009	1.5773	-0.9101	Br
5 Br5	0.6006	-1.5770	-0.9108	Br
6 C6	8.3941	-1.0382	-0.5997	C
7 C7	7.0108	-1.0548	-0.6092	C
8 C8	6.3236	0.0001	-0.0000	C
9 C9	7.0107	1.0550	0.6091	C
10 C10	8.3940	1.0386	0.5995	C
11 C11	9.1010	0.0002	-0.0001	C
12 H12	6.4870	-1.8731	-1.0818	H
13 H13	6.4868	1.8733	1.0816	H
14 C14	4.8937	0.0000	0.0000	C
15 N15	3.7200	0.0000	0.0000	N
16 F16	9.0847	-2.0259	-1.1700	F
17 F17	10.4289	0.0003	-0.0001	F
18 F18	9.0845	2.0264	1.1699	F

@<TRIPOS>BOND
1 1 2 1
2 1 3 1
3 1 4 1
4 1 5 1
5 6 7 1
6 6 11 1
7 6 16 1
8 7 8 1
9 7 12 1
10 8 9 1
11 8 14 1
12 9 10 1
13 9 13 1
14 10 11 1
15 10 18 1
16 11 17 1

17 14 15 1

$F\text{-}C\text{-}I_3\cdots NC\text{-}C_6H_2\text{-}F_3$

Molecule Name
18 17
SMALL
NO_CHARGES

@<TRIPOS>ATOM
1 C1 -0.0000 0.0000 0.0000 C
2 F2 -1.3562 -0.0000 -0.0000 F
3 I3 0.6571 0.0000 2.0380 I
4 I4 0.6572 -1.7649 -1.0191 I
5 I5 0.6572 1.7650 -1.0189 I
6 C6 8.4241 0.0001 -1.1990 C
7 C7 7.0408 0.0001 -1.2181 C
8 C8 6.3536 0.0001 0.0000 C
9 C9 7.0407 0.0002 1.2182 C
10 C10 8.4240 0.0003 1.1992 C
11 C11 9.1310 0.0002 0.0001 C
12 H12 6.5170 0.0000 -2.1630 H
13 H13 6.5168 0.0002 2.1631 H
14 C14 4.9237 -0.0000 0.0000 C
15 N15 3.7500 -0.0000 0.0000 N
16 F16 9.1147 0.0001 -2.3395 F
17 F17 10.4589 0.0003 0.0002 F
18 F18 9.1145 0.0003 2.3398 F
@<TRIPOS>BOND
1 1 2 1
2 1 3 1
3 1 4 1
4 1 5 1
5 6 7 1
6 6 11 1
7 6 16 1
8 7 8 1
9 7 12 1
10 8 9 1
11 8 14 1
12 9 10 1
13 9 13 1
14 10 11 1
15 10 18 1
16 11 17 1

17 14 15 1

F-C-Cl_3···NCF

```
Molecule Name
8 6
SMALL
NO_CHARGES


@<TRIPOS>ATOM
1 C1    -25.3146    0.0329    2.3815 C
2 F2    -23.9680    0.0268    2.4014 F
3 Cl3   -25.8529    1.6908    2.1432 Cl
4 Cl4   -25.8490   -0.9918    1.0551 Cl
5 Cl5   -25.8895   -0.5927    3.9220 Cl
6 N6    -28.9097    0.0492    2.3281 N
7 C7    -30.0818    0.0546    2.3107 C
8 F8    -31.3496    0.0604    2.2918 F
@<TRIPOS>BOND
1 1 2 1
2 1 3 1
3 1 4 1
4 1 5 1
5 6 7 3
6 7 8 1
```

F-C-Br_3···NCF

Molecule Name
8 6
SMALL
NO_CHARGES

@<TRIPOS>ATOM
1 C1 -10.6860 -0.1171 1.1796 C
2 F2 -9.3406 -0.1298 1.0908 F
3 Br3 -11.2372 -1.7532 2.0107 Br
4 Br4 -11.3925 0.0224 -0.5960 Br
5 Br5 -11.1925 1.3959 2.2404 Br
6 N6 -14.2439 -0.0838 1.4143 N
7 C7 -15.4138 -0.0730 1.4914 C
8 F8 -16.6791 -0.0614 1.5757 F
@<TRIPOS>BOND
1 1 2 1
2 1 3 1
3 1 4 1
4 1 5 1
5 6 7 3
6 7 8 1

F-C-I_3···NCF

Molecule Name
8 6
SMALL
NO_CHARGES

@<TRIPOS>ATOM
1 C1 -24.8447 -0.1174 2.2723 C
2 F2 -23.4882 -0.1891 2.2534 F
3 I3 -25.5704 -1.3357 0.6688 I
4 I4 -25.3787 1.9376 2.0009 I
5 I5 -25.4959 -0.8532 4.1741 I
6 N6 -28.4190 0.0718 2.3219 N
7 C7 -29.5900 0.1337 2.3380 C
8 F8 -30.8560 0.2008 2.3554 F
@<TRIPOS>BOND
1 1 2 1
2 1 3 1
3 1 4 1
4 1 5 1
5 6 7 3
6 7 8 1
